# The Diagnostic Value of Neuron-Specific Enolase in Children with Mild Blunt Trauma Requiring Cranial CT Scan

**DOI:** 10.2147/OAEM.S223179

**Published:** 2020-01-13

**Authors:** Javad Mozafari, Hassan Motamed, Mohammad Ghasem Hanafi, Bita Fatehifar

**Affiliations:** 1 Department of Emergency Medicine, Golestan Hospital, Ahvaz Jundishapur University of Medical Sciences, Ahvaz, Iran; 2 Department of Radiology, Ahvaz Golestan Hospital, Ahvaz Jundishapur University of Medical Sciences, Ahvaz, Iran; 3 Student Research Committee, Ahvaz Jundishapur University of Medical Sciences, Ahvaz, Iran

**Keywords:** head trauma, blunt trauma, diagnosis, neuron-specific enolase, children

## Abstract

**Background:**

The present study was conducted to investigate the relationship between serum levels of enolase and pathological findings obtained from CT scans of the brain in children with mild blunt brain trauma and help with a more accurate diagnosis of brain injuries.

**Methods:**

The present observational study was conducted on children presenting with head traumas to the emergency department (ED) of Golestan Hospital in Ahvaz, Iran in 2016. A venous blood sample was immediately taken by the ward nurse from all the eligible patients within 6 hrs of the incident after obtaining their information, performing initial examinations and their initial stabilization. Laboratory serum levels and the corresponding interpretations of CT scans of the brain were collected, recorded and then evaluated and analyzed.

**Results:**

A total of 62 children with mild blunt brain trauma were included in the study. A significant difference was observed between the positive CT scan group (2.7±9.74 µg/L) and the negative group (4.23±1.33 µg/L) in terms of serum levels of enolase (P<0.0001). The area under the receiver operating characteristic (ROC) curve was 0.992 for serum levels of enolase in diagnosing brain lesions caused by mild head traumas. Moreover, with a cut-off point of 6.97 µg/L, brain lesions could be detected with a sensitivity of 93.55% and a specificity of 100%.

**Conclusion:**

Serum levels of enolase were found to be higher in patients with brain injuries. This highly accurate diagnostic biomarker can be recommended for estimating the presence of brain lesions associated with mild head traumas in infants.

## Introduction

Traumatic brain injuries (TBIs) caused by an external pressure on the brain can temporarily or permanently disrupt the nervous function.^1^ This damage is the reason for hundreds of thousands of presentation to emergency departments (EDs) each year.^2^ Accidents are a major cause of presenting to EDs and the leading cause of death in children below the age of 15 years. Falls (32%) and road traffic accidents (31.1%) constitute the main causes of trauma, and head (38.5%), multiple traumas (34.4%) and limbs (18.9%) are the most common anatomic sites of the injuries.^3^ Despite the preventive measures taken, TBIs still remain a major cause of morbidity in children, annually afflicting 100,000–150,000 children. Although the majority of these injuries are mild, and do not require hospitalization, 10–15% are severe and can cause significant damage or death.^4^,^5^

The mechanism of damage to the head varies with age. The damage caused by impacts is a major injury in infants and is accompanied by severe diffuse injury if repeated. Delays in treating these injuries are usually associated with hypoxic ischemia, which distinguishes it from other head traumas, and generally causes the worst outcomes in these patients compared to other cases of head trauma.^5^ Falls are the most dominant mechanism of damage in toddlers, whereas damage caused by road traffic accidents is the most prevalent in adults.^6^

Non-penetrating head traumas are mostly mild, leading to a GCS score of 14–15. Standard CT scans of the brain are required for determining brain damage based on the symptoms of the injured.^7^ Performing CT scan exposes the patient to X-ray, which can increase the risk of cancers. The risk of leukemia and brain tumors is higher in children who undergo numerous CT scans up to the age of 15 years.^8^ Research suggests that one case of leukemia and one case of brain tumor can be expected within 10 years of CT scan in every 10,000 children below the age of 10 years undergoing CT scan. The present study was conducted to help more accurately diagnose brain injuries by investigating the relationship between serum levels of neuron-specific enolase (NSE) and pathological findings obtained from CT scans of the brain in children with mild blunt brain trauma.

## Materials and Methods

### Study Design

This study had been conducted according to the Standards for Reporting Diagnostic Accuracy (STARD).^9^

After obtaining ethics code from the ethics committee of Jundishapur University as per the ethical principles stated for medical research involving human subjects in the Declaration of Helsinki, the present observational study was conducted on all children with head traumas presenting to the ED of Golestan Hospital in Ahvaz in 2016.

### Participants

After the initial examinations and stabilization of the patients with TBIs by a senior emergency medicine resident, CT scans of the brain were performed according to the latest guidelines in case the indications appeared, including an age of 6 months to 18 years, a GCS score of 14 and 15, the mechanism of damage being of the type of traffic accidents and domestic or sport injuries, the incident occurring within the previous 6 hrs, the parents giving consent for the participation of their children in the study, lack of pregnancy, no history of alcohol or drug abuse, no history of neurological diseases such as seizure and epilepsy and the absence of severe road traffic injuries such as overturned vehicle or being thrown out of the car.

The exclusion criteria comprised a history or clinical evidence for stroke, cerebral hemorrhage, head trauma and infection of central nervous system within the previous 3 months, a history of brain tumors, having injuries other than mild brain trauma such as limb fractures, having a history of major diseases such as diabetes, heart problems and asthma, a BMI below the fifth percentile or above the 95th percentile, severe traffic injuries such as overturned vehicle or being thrown out of the car.

### Test Methods

In the present analytical epidemiological study, a venous blood sample was immediately taken by the ward nurse from all the eligible patients within 6 hrs of the incident after obtaining their information, performing initial examinations and their initial stabilization. The patients were then referred to an imaging unit for cranial CT scan. The initial data recorded in relevant forms involved the mechanism of injury, the presence of lacerations, scratches, contusions and the size and site of the lesion in the scalp and face, GCS score upon examination, headache, nausea, vomiting, dizziness, neurological defects, amnesia, reductions in levels of consciousness and their duration. The collected samples were then immediately transferred to the central laboratory of Golestan Hospital, centrifuged and their serum was separated and kept at −80ºC. After collecting the required number of samples, laboratory kits for measuring the biomarker of enolase (CanAg Humn NSE ELI, made in Switzerland) were used to individually determine and record the serum concentration of enolase in each sample according to the ELISA method without any knowledge of the results of CT scans of the brain. The initial CT scans of the brain of all the patients were performed by a CT scan machine, the results interpreted by an emergency medicine specialist, and the films subsequently interpreted independently by one neuro-radiologist, who was unaware of the results of enolase levels. The patients were dealt with according to the clinical protocol of handling head-trauma patients. After collecting and recoding the results of laboratory serum levels and the corresponding interpretations of CT scans of the brain, one group of the patients with positive-for-trauma pathological findings in CT scans and the other group with negative findings were analyzed based on the calculated sample size.

### Analysis

Statistical analyses were performed in SPSS. The normally distributed data were expressed as Mean ± Standard Deviation (SD). The Chi-square test was used for the qualitative data, the paired *t*-test for the normally distributed quantitative data, and Mann–Whitney *U*-test for the non-standard quantitative data. The ROC curve was plotted for the ability of NSE to diagnose mild blunt brain trauma in children and predict hospital mortality, and cut-off points with the highest sensitivity and specificity were determined. P<0.05 was set as the level of statistical significance.

## Results

Sixty-two children with mild blunt brain traumas were studied in two groups of 31, namely the positive and negative CT scan groups. Table 1 presents the patients’ demographic information, clinical symptoms and GCS scores.

**Table 1:** Frequency and Mean Distribution of Demographic and Clinical Variables of Patients According to Two Groups

		CT Positive	CT Negative	P-value
Age, year Mean ± SD		8.57 ± 5.16	8.32 ± 4.72	0.484
Gender, Male N (%)		22 (71)	24 (77.4)	0.562
Serum NSE, µg/L Mean ± SD		9.74 ± 2.7	4.23 ± 1.33	P < 0.001
GCS N (%)	14	17 (54.8)	6 (19.4)	0.004
15	14 (45.2)	25 (80.6)
Headache N (%)	Yes	19 (61.3)	14 (45.2)	0.126
No	11 (35.5)	17 (54.8)
Vertigo N (%)	Yes	7 (22.6)	3 (9.7)	0.301
No	24 (77.4)	28 (90.3)
Confusion N (%)	Yes	2 (6.5)	0	0.492
No	29 (93.5)	31 (100)

**Abbreviation:** NSE, neuron-specific enolase.

Serum levels of enolase were found to be 9.74±2.7 µg/L in the positive CT scan group and 4.23±1.33 µg/L in the negative group, suggesting a statistically significant difference (P<0.0001). In the positive CT scan group, the frequency of a GCS score of 14 was 17 (54.8%) and that of a GCS score of 15 was 14 (45.2%), and in the negative CT scan group, these frequencies were, respectively, 6 (19.4%) and 25 (80.6%), suggesting a statistically significant difference (P=0.004) (Table 1).

**Figure 1:**
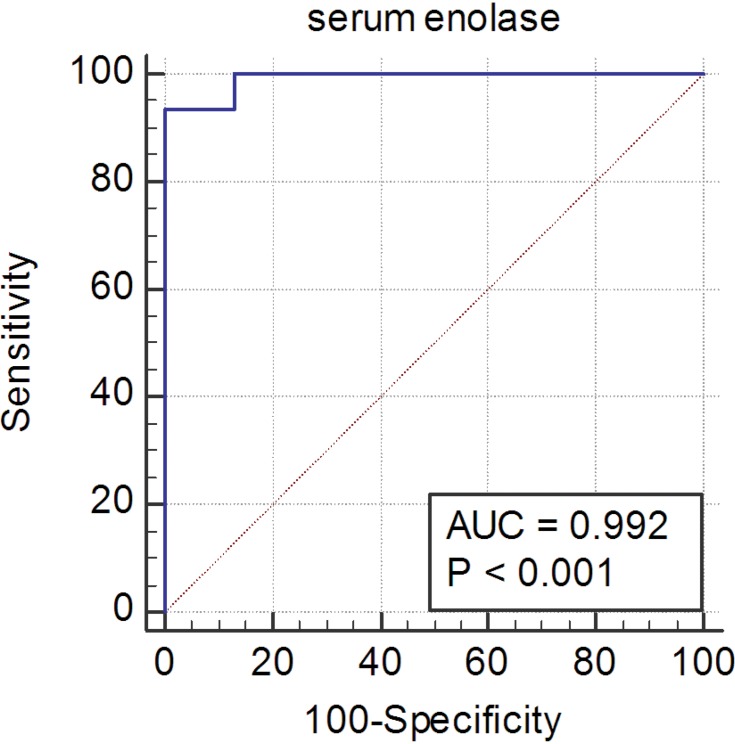
Receiver operating characteristic (ROC) curve for the diagnosis of NSE.

The area under the ROC curve for serum levels of enolase was found to be 0.992 (SE=0.001, P<0.007) in diagnosing brain lesions caused by mild head traumas (Table 2) (Figure 1). The optimal cut-off points obtained also included 5.74 µg/L with a sensitivity of 100% and a specificity of 87.1%, and 6.97 µg/L with a sensitivity of 93.55% and a specificity of 100%.

**Table 2:** Correlation with ROC and Characteristics Factors

		AUC	SE	CI 95%	P-value
Overall		0.992	0.007	(0.927, 1)	<0.0001
Gender	Male	0.985	0.013	0.896, 1)	<0.0001
Female	1	0	(0.794, 1)
Age	(0–2)	–	–	–	<0.0001
	(2–10)	1	0	(0.910, 1)	
(10–18)	0.954	0.039	(0.777, 0.999)
GCS	14	0.980	0.024	(0.818, 1)	<0.0001
15	1	0	(0.910, 1)
Headache	Yes	0.979	0.019	(0.859, 1)	<0.0001
No	1	0	(0.877, 1)	<0.0001
Vertigo	Yes	1	0	(0.692, 1)	<0.0001
No	0.994	0.007	(0.920, 1)	<0.0001

## Discussion

Head and neck traumas caused by accidents are common reasons for presenting to EDs.^10^ CT scan is an effective technique available to physicians for diagnosing head lesions;^11^ nevertheless, it is costly, and causes a longer hospital stay, and exposes patients to the risks of radiation.

The present findings showed significantly higher serum levels of enolase in the children with mild head traumas and positive results of CT scans of the brain, ie, 9.74±2.7 µg/L, compared to in another group of these children with negative CT scans (4.23±1.33 µg/L). In contrast, de Kruijl et al (2001) found serum levels of enolase to be 9.8 µg/L in patients with mild head traumas and 9.4 µg/L in healthy patients, suggesting that brain trauma causes a negligible increase in the levels of this enzyme.^12^ According to the present study results, in the positive CT scan group, the frequency of a GCS score of 14 was 54.8% and that of a GCS score of 15 was 45.2%, and in the negative CT scan group, these frequencies were, respectively, 19.4% and 80.6%, suggesting higher levels of consciousness or a GCS score of 15 in the negative group. Initial levels of consciousness based on the GCS score are a deciding factor for initial treatments and long-term complications.

According to the present study results, with a cut-off point of 5.74µg/L, serum levels of enolase were found to diagnose intracranial lesions with a sensitivity of 100% and a specificity of 87.1%. Moreover, with a cut-off point of 6.97µg/L, serum levels of enolase could be used to diagnose these lesions with a sensitivity of 93.5% and a specificity of 100%. These findings are consistent with positive CT scan results in girls and boys aged 2–10 years and 10–18 years. Meric et al (2008) investigated head traumas and changes in serum levels of enolase, and reported an area under the ROC curve of 0.931 with a cut-off point of 20.52 µg/L for serum levels of enolase and a sensitivity of 87% and a specificity of 82.1%,^13^ which is inconsistent with the present research.

Bandyopadhyay et al (2005) investigated serum levels of enolase in patients suspected of head traumas, and found these levels to be a predictor of the GCS score after TBIs,^14^ which is consistent with the present findings considering the area under the ROC curve.

Similar to the present study, Fridriksson et al (2000) found serum levels of enolase to be a predictor of intracranial lesions in a positive and a negative CT scan group, although they argued that this method should not be individually considered a method of diagnosing head and neck traumas given the low sensitivity obtained, ie, a cut-off point of 15.3 µg/L for serum levels of NSE with a sensitivity of 77% and a specificity of 52%.^15^ In addition, they observed no significant differences between the positive and negative CT scan groups in terms of nausea, vomiting, headache, dizziness and contusion.

## Conclusion

Serum levels of enolase were higher in patients with brain injuries. Furthermore, this highly accurate diagnostic biomarker can be recommended for identifying brain lesions associated with mild head traumas in infants. The cut-off point of 6.97 µg/L was also associated with a higher sensitivity and specificity compared to the findings of other studies.

## References

[1] Young LA, Rule GT, Bocchieri RT, Burns JM. Biophysical mechanisms of traumatic brain injuries. *Semin Neurol*. 2015;35(1):5–11. doi:10.1055/s-00000071 25714862

[2] Weil ZM, Karelina K. Traumatic brain injuries during development: implications for alcohol abuse. *Front Behav Neurosci*. 2017;11:135. doi:10.3389/fnbeh.2017.00135 28775682 PMC5517445

[3] Kemp A, Sibert J. Childhood accidents: epidemiology, trends, and prevention. *J Accid Emerg Med*. 1997;14(5):316–320. doi:10.1136/emj.14.5.316 9315935 PMC1343099

[4] Blennow K, Brody DL, Kochanek PM, et al. Traumatic brain injuries. *Nat Rev Dis Primers*. 2016;2:16085. doi:10.1038/nrdp.2016.85 27853132

[5] Dayan PS, Holmes JF, Schutzman S, et al. Risk of traumatic brain injuries in children younger than 24 months with isolated scalp hematomas. *Ann Emerg Med*. 2014;64(2):153–162. doi:10.1016/j.annemergmed.2014.02.003 24635991

[6] Kamboj A, Chounthirath T, Xiang H, Smith GA. Traumatic brain injuries associated with consumer products at home among US children younger than 5 years of age. *Clin Pediatr (Phila)*. 2017;56(6):545–554. doi:10.1177/0009922816664064 27600615

[7] Marx J, Walls R, Hockberger R. *Rosen’s Emergency Medicine-Concepts and Clinical Practice E-Book*. Elsevier Health Sciences; 2013.

[8] Asl JF, Shirbandi K, Naghashpour M, Mehr FJ. Medical interns’ and residents’ awareness of radiation protection in pediatric diagnostic imaging. *Jentashapir J Health Res*. 2018;9(1).

[9] Bossuyt PM, Reitsma JB, Bruns DE, et al. STARD 2015: an updated list of essential items for reporting diagnostic accuracy studies. *BMJ*. 2015;351:h5527. doi:10.1136/bmj.h5527 26511519 PMC4623764

[10] Kanwar R, Delasobera BE, Hudson K, Frohna W. Emergency department evaluation and treatment of cervical spine injuries. *Emerg Med Clin North Am*. 2015;33(2):241–282. doi:10.1016/j.emc.2014.12.002 25892721

[11] Kulvatunyou N, Lees JS, Bender JB, Bright B, Albrecht R. Decreased use of cervical spine clearance in blunt trauma: the implication of the injury mechanism and distracting injury. *Accid Anal Prev*. 2010;42(4):1151–1155. 20441825 10.1016/j.aap.2009.12.029

[12] de Kruijk JR, Leffers P, Menheere PP, Meerhoff S, Twijnstra A. S-100B and neuron-specific enolase in serum of mild traumatic brain injury patients. A comparison with health controls. *Acta Neurol Scand*. 2001;103(3):175–179. doi:10.1034/j.1600-0404.2001.103003175.x 11240565

[13] Meric E, Gunduz A, Turedi S, Cakir E, Yandi M. The prognostic value of neuron-specific enolase in head trauma patients. *J Emerg Med*. 2010;38(3):297–301. doi:10.1016/j.jemermed.2007.11.032 18499387

[14] Bandyopadhyay S, Hennes H, Gorelick MH, Wells RG, Walsh-Kelly CM. Serum neuron-specific enolase as a predictor of short-term outcome in children with closed traumatic brain injury. *Acad Emerg Med*. 2005;12(8):732–738. doi:10.1197/j.aem.2005.02.017 16079426

[15] Fridriksson T, Kini N, Walsh-Kelly C, Hennes H. Serum neuron-specific enolase as a predictor of intracranial lesions in children with head trauma: a pilot study. *Acad Emerg Med*. 2000;7(7):816–820. doi:10.1111/acem.2000.7.issue-7 10917333

